# Nutrient Digestibility and Growth Performance of Juvenile *Penaeus vannamei* Fed Diets Containing the Filamentous Fungus, *Paecilomyces variotii*, Produced From Corn Ethanol Thin Stillage

**DOI:** 10.1155/anu/6937869

**Published:** 2026-05-30

**Authors:** Alberto J. P. Nunes, Marcelo Pereira de Aguiar Toledo, Luana Quisini Vivan, Daniele Ferreira Marques, Caio G. D. Gomes

**Affiliations:** ^1^ LABOMAR – Instituto de Ciências do Mar, Universidade Federal do Ceará, Avenida da Abolição, 3207 – Meireles, Fortaleza, 60.165-081, Ceará, Brazil, ufc.br; ^2^ Ideaqua Consultoria, Av. Egisto Sicchieri, 964, Casa 301, Sertãozinho, 14.161-000, São Paulo, Brazil; ^3^ FS Indústria de Biocombustíveis Ltda., Distrito Industrial Senador Atílio Fontana, Lucas do Rio Verde, 78.455-000, Mato Grosso, Brazil

**Keywords:** fish meal replacement, microbial protein, mycoprotein, nutrient bioavailability

## Abstract

Fungal proteins are emerging ingredients having high potential for use in aquafeeds, yet digestibility and practical inclusion limits remain unclear for marine shrimp. This study evaluated a filamentous fungus (*Paecilomyces variotii [PV]*) produced from corn ethanol thin stillage as a replacement for salmon by‐product meal (SLM) and poultry by‐product meal (PBM) in diets for juvenile *Penaeus vannamei*. Five diets were formulated by replacing SLM and PBM with PV (0%–100% replacement; PV from 0% to 22.60% of the diet). After 78 days, shrimp performance was similar across treatments. Survival remained high (95.6 ± 3.4%). Weekly growth averaged 1.04 ± 0.08 g/week, with a gained yield of 1168 ± 92 g/m^2^. Apparent feed intake (AFI) was 14.6 ± 0.6 g/shrimp, and feed conversion ratio (FCR) was 1.33 ± 0.09. Final body weight (BW) differed statistically among dietary treatments, with shrimp fed the diet containing 22.60% PV exhibiting a reduction (12.31 ± 1.89 g) compared with 0% PV (12.80 ± 2.02 g). In a digestibility assay (substitution method; 30% test ingredient), ingredient‐level crude protein (CP) apparent digestibility coefficient (ADC) was higher for SLM (72.02 ± 1.96%) than for the PV (62.28 ± 0.48%). Amino acid (AA) digestibility was consistently lower for PV, with marked reductions in methionine (Met) (73.21 ± 1.72% vs. 85.71 ± 1.05%) and lysine (Lys) (86.23 ± 0.66% vs. 89.35 ± 1.10%) compared with SLM. Whole‐body proximate composition at harvest was largely unaffected by dietary treatment, whereas nutrient and energy retention differed, with the highest protein retention observed at 75% replacement and reduced lipid and energy retention at 100% replacement. Overall, results indicated that PV can replace up to 75% of SLM and PBM, corresponding to 16.95% dietary inclusion, in diets for *P. vannamei* without impairing growth performance, whereas complete replacement (22.60% PV) reduces nutrient utilization efficiency and final BW.

## 1. Introduction

The continued expansion of shrimp aquaculture worldwide has substantially increased the demand for traditional protein sources, intensifying pressure on both marine and terrestrial resources [1–3]. In particular, the continued reliance on fish meal derived from wild forage fisheries highlights ecological and supply limitations associated with marine resource use [4]. In contrast, increased use of land‐based protein sources, including crop ingredients and terrestrial animal by‐products, raises concerns related to land use, resource demand, and associated biodiversity trade‐offs [2, 3]. To sustain the growth of the aquaculture industry while addressing environmental, economic, and supply constraints, there is a growing need to develop nonconventional and sustainable protein sources capable of supporting shrimp performance without compromising feed efficiency or nutrient utilization. Among the emerging alternatives, ingredients produced through microbial fermentation have attracted increasing interest due to their scalability, compositional consistency, and potential integration into circular bioeconomy systems [5–8].

Proteins derived from yeasts, bacteria, and microalgae have been investigated in shrimp feeds as alternative protein ingredients to replace fish meal [7]. Yeast‐based ingredients evaluated for fish meal substitution have included brewer’s yeast [9], yeast extract [10], yeast hydrolysate [11], and yeast‐derived proteins produced via fermentation [12]. Microalgal single–cell proteins have also been tested as fish meal and soybean meal replacers in shrimp diets, including *Chlorella vulgaris*, *Spirulina platensis*, and *Scenedesmus* sp. meals, with studies reporting variable responses depending on the inclusion level and processing method [13–16]. In addition, bacterial proteins have been evaluated as fish meal substitutes in *Penaeus vannamei* diets, including methanotrophic (*Methylococcus capsulatus* [15, 17–19]), acetogenic (*Clostridium autoethanogenum* [15]), and photosynthetic bacterial meals (*Rhodobacter sphaeroides*; [20]). These ingredients can deliver concentrated protein utilizing industrial side streams while reducing reliance on traditional protein sources.

Fungal‐derived proteins have been widely studied in fish feeds, demonstrating that filamentous fungi biomass can effectively replace conventional protein sources while maintaining growth performance. In Atlantic salmon, *Salmo salar*, *Paecilomyces variotii* (PV) inclusion at up to 20% of dietary protein reduced apparent digestibility of crude protein (CP) and energy but did not impair growth and improved feed conversion ratio (FCR) and nutrient retention efficiency [21]. Earlier work in rainbow trout, *Oncorhynchus mykiss*, also reported reduced digestibility at higher inclusion levels (20%–30%), although moderate inclusion appeared to mitigate intestinal inflammation and support gut health [22]. Complementary studies with Atlantic salmon pre‐ and postsmolts showed that PV modulates immune responses, gut morphology, and microbiota composition, with evidence of cytokine regulation, enhanced immunoglobulin production, and alterations in mucosal health depending on the inclusion level [23, 24]. In addition, recent work demonstrated that increasing dietary inclusion of PV reduces environmental impacts of salmon production, including greenhouse gas emissions, reliance on marine resources (eFIFO), and food–feed competition, supporting its role as a sustainable protein source in aquafeeds [25]. Despite the substantial body of evidence generated in fish species, the nutritional value and practical inclusion limits of fungal proteins in shrimp diets remain poorly defined, thereby constraining their effective application in commercial shrimp feed formulations.

The present study evaluated the filamentous fungus PV produced from corn ethanol thin stillage as a replacement for salmon by‐product meal (SLM) and poultry by‐product meal (PBM) in diets for juvenile *P. vannamei*. Growth performance, apparent digestibility of CP and amino acids (AAs), and whole‐body nutrient and energy retention were assessed under controlled clear water conditions. The objective was to define the practical inclusion limits of this PV for whiteleg shrimp (*P. vannamei*) feeds and to elucidate the nutritional mechanisms underlying shrimp responses to increasing levels of animal protein replacement.

## 2. Materials and Methods

### 2.1. Experimental Design

The study was conducted in two experimental phases to evaluate the effects of the dietary inclusion of PV on the growth performance of juvenile *P. vannamei*. The first phase assessed the growth performance and feed utilization in shrimp‐fed diets with graded inclusion levels of PV. The second phase determined the apparent digestibility coefficients (ADCs) for CP and AAs of PV in comparison to SLM.

In the growth performance trial, five isonitrogenous and isolipidic diets were formulated by progressively replacing SLM and PBM with PV. A diet without any PV was used as a control (CTL). Juvenile shrimp were stocked in indoor tanks (0.5 m^3^) at a density of 105 shrimp/m^2^, with 10 replicate tanks per dietary treatment. The nutrient digestibility assay was conducted in an indoor clear water recirculating system using rectangular tanks. Three experimental diets were evaluated, including a reference (REF) diet and two test diets containing 30% PV or 30% SLM. Juvenile shrimp were stocked at a density of 63 shrimp/m^2^, with 15 replicates per dietary treatment. ADCs were determined using an indirect marker method.

### 2.2. Fungal Biomass

The fungal biomass was produced by FS—Fueling Sustainability (Lucas do Rio Verde, Brazil) under a license from Enifer Oy (Finland). For the present study, the fungal biomass was produced from PV using thin stillage derived from corn‐based ethanol production as the primary fermentation substrate. Thin stillage is the clarified liquid fraction obtained after distillation and removal of solids during the dry‐grind corn ethanol process and is characterized by high concentrations of soluble organic matter and minerals, with low residual protein and lipid contents.

A sample of 20.1 kg of PV was used in this study. The fungal biomass was delivered as brown pellets, which were pooled and ground using a centrifugal mill (5 hp; model MCS 280, Máquinas Vieira Indústria e Comércio Ltda., Tatuí, Brazil) equipped with a 300‐µm screen to standardize particle size prior to diet manufacturing and chemical analyses. SLM was used as a REF marine protein. The ingredient was produced from Atlantic salmon (*S. salar*) processing by‐products by the Fiordo Austral Group (Puerto Montt, Chile).

The fungal biomass contained 51.72% CP (as‐is basis), 2.54% ether extract (EE), and 11.66% ash, with a relatively high crude fiber (CF) content (8.66%), reflecting the presence of fungal cell‐wall polysaccharides (Table 1). The ingredient exhibited a low lipid‐derived energy contribution, resulting in a gross energy (GE) of 14.89 MJ/kg and negligible calcium content (0.0041%), while phosphorus reached 1.80%. The AA profile showed a total essential AA (ΣEAA) content of 18.13%, with lysine (Lys) (2.76%) and methionine (Met) (0.68%) being lower than the levels observed in the SLM. The SLM exhibited a higher nutritional density, with 67.08% CP and 10.88% EE, resulting in a higher GE concentration (19.00 MJ/kg). Ash content reached 14.07%, accompanied by elevated levels of calcium (3.22%) and phosphorus (2.20%), consistent with the presence of bone material. SLM presented a superior AA profile, with a ΣEAA content of 24.58%, notably higher concentrations of Lys (4.47%), Met (1.69%), and sulfur AAs (Met + cysteine, 2.35%), supporting its use as a benchmark high‐quality marine protein source for penaeid shrimp.

**Table 1:** Proximate composition, amino acid profile (%, as‐is basis), and gross energy (MJ/kg) of the filamentous fungi, *Paecilomyces variotii*, and salmon by‐product meal (SLM).

Nutrient/ingredient	Composition (%, as‐is)		CV (%)
	*P. variotii*	SLM	
Dry matter	92.20	92.45	0.19
Crude protein	51.72	67.08	18.28
Ether extract	2.54	10.88	87.89
Ash	11.66	14.07	13.25
Crude fiber	8.66	0.13	137.24
Calcium	0.0041	3.22	141.06
Phosphorous	1.80	2.20	14.14
Gross energy (MJ/kg)	14.89	19.00	17.16
Essential amino acids (EAAs)^a^			
Arginine	3.32	3.74	8.41
Histidine	0.93	1.66	39.86
Isoleucine	1.74	2.07	12.25
Leucine	3.01	3.62	13.01
Lysine	2.76	4.47	33.45
Methionine (Met)	0.68	1.69	60.27
Met + cysteine	1.06	2.35	53.50
Phenylalanine	1.62	2.21	21.79
Threonine	2.00	2.45	14.30
Valine	2.07	2.67	17.90
Nonessential amino acids (NEAAs)			
Alanine	2.75	4.27	30.62
Aspartic acid	3.57	5.66	32.02
Cysteine	0.38	0.66	38.07
Glycine	2.23	6.84	71.88
Glutamic acid	4.88	8.10	35.08
Hydroxyproline	0.01	1.55	139.61
Proline	1.72	3.64	50.66
Serine	1.96	2.74	23.47
Taurine	0.01	0.86	138.17
Tyrosine	1.36	1.59	11.03
∑EAA^a^	18.13	24.58	21.36
∑NEAA	18.87	35.91	43.99
∑EAA^a^ + NEAA	37.00	60.49	34.08

Abbreviation: CV, coefficient of variation.

^a^Except tryptophan.

### 2.3. Diet Formulation and Manufacture

Digestibility was evaluated using one REF diet and two test diets formulated according to the conventional 70:30 substitution method. The REF diet was formulated to meet the nutritional requirements of juvenile *P. vannamei* and served as the basal mixture for the preparation of the test diets (Table 2). The experimental diets were produced by replacing 30% of the REF diet with the ingredient under evaluation, resulting in (1) an SLM diet, containing 70% REF diet and 30% SLM; and (2) a PV diet, containing 70% REF diet and 30% PV. Chromic oxide III (Cr_2_O_3_) was incorporated at 1.00% (as‐is basis) into the REF and both test diets as an inert marker for apparent digestibility determination.

**Table 2:** Ingredient, proximate, and amino acid composition (%, as‐is basis) of the experimental diet and reference mixture (REF) used for the in vivo digestibility evaluation of *Paecilomyces variotii* (PV) and SLM in juvenile *P. vannamei*.

Ingredients	Diet/inclusion (% of the diet, as‐is)		
	REF	SLM	PV
Soybean meal^a^	30.00	21.00	21.00
Wheat flour^b^	23.80	16.66	16.66
Salmon by‐product meal (SLM)^c^	22.00	45.40	15.40
*P. variotii^c^*	—	—	30.00
Salmon oil^d^	3.90	2.73	2.73
Wheat gluten meal^e^	2.50	1.75	1.75
Soy lecithin	0.67	0.47	0.47
Calcium carbonate	2.27	1.59	1.59
Krill meal^f^	8.00	5.60	5.60
MSF^g^	2.42	1.69	1.69
Salt	1.00	0.40	0.40
Chromic oxide III^h^	1.00	1.00	1.00
Synthetic binder^i^	0.50	0.35	0.35
Vitamin‐mineral premix^j^	1.00	0.70	0.70
Cholesterol^k^	0.10	0.07	0.07
Choline chloride, 60%^l^	0.30	0.21	0.21
Stay C, 35%^m^	0.29	0.20	0.20
DL‐methionine, 99%^n^	0.25	0.18	0.18
Proximate composition (%, as‐is)			
Dry matter	95.70	95.62	95.46
Crude protein	40.75	48.75	44.92
Ether extract	11.95	9.15	9.06
Crude fiber	1.50	1.00	1.02
Ash	13.55	14.52	10.88
Gross energy (MJ/kg)	18.37	18.74	18.10
Calcium	1.90	2.63	1.46
Phosphorous	1.51	1.95	1.65
Essential amino acid (EAA)			
Arginine	2.47	2.90	2.81
Histidine	1.09	1.31	0.97
Isoleucine	1.63	1.80	1.76
Leucine	2.78	3.06	2.89
Lysine	2.05	2.78	2.35
Methionine (Met)	0.92	1.14	0.84
Met + cysteine	1.36	1.60	1.37
Phenylalanine	1.71	1.83	1.64
Threonine	1.46	1.74	1.61
Valine	1.78	2.05	1.87
Nonessential amino acids (NEAAs)			
Alanine	2.09	2.78	2.35
Aspartic acid	3.50	3.98	3.58
Cysteine	0.44	0.46	0.53
Glycine	2.69	3.96	2.55
Glutamic acid	6.73	6.91	6.21
Hydroxyproline	0.41	0.78	0.01
Proline	2.50	2.82	2.24
Serine	1.76	2.03	1.83
Taurine	0.26	0.41	0.20
Tyrosine	1.23	1.38	1.33
∑EAA^o^	16.29	19.03	17.31
∑NEAA	20.37	24.15	19.50
∑EAA^o^ + NEAA	36.66	43.18	36.81
Chromium (mg/kg, DM)	3.45 ± 2.35	3.40 ± 2.37	3.46 ± 2.04

*Note:* Chromium content (mean ± SD) expressed on a dry matter (DM) basis (mg/kg).

^a^Bunge Alimentos S.A. (Luiz Eduardo Magalhães, Brazil). 8.55% moisture, 47.57% CP (crude protein), 1.82% EE (ether extract), 3.02% CF (crude fiber), 6.86% ash, 0.61% methionine (Met), 2.92% lysine (Lys), and 1.28% Met + cysteine (M + C).

^b^12.68% moisture, 12.44% CP, 1.55% EE, 0.37% CF, 0.81% ash, 0.21% Met, 0.28% Lys, and 0.61% M + C.

^c^Check Table 1 for nutrient composition.

^d^Fiordo Austral Group (Puerto Montt, Chile). 99.52% total lipids, 18.34% LOA, 5.70% LNA, 0.26% ARA, 3.25% EPA, 4.12% DHA, 13.60% total n‐3 PUFA, and 19.98% total n‐6 PUFA.

^e^Agridient, Inc. (Farmington Hills, USA). 9.09% moisture, 79.19% CP, 3.96% EE, 0.18% CF, 0.82% ash, 1.24% Met, 1.33% Lys, and 2.68% M + C.

^f^QRILL Aqua, The Qrill Company AS (Lysaker, Norway). 6.47% moisture, 57.42% CP, 22.00% EE, 3.51% CF, 12.23% ash, 1.57% Met, 4.00% Lys, and 1.98% M + C.

^g^Monobasic sodium phosphate. 0.60% calcium, 20.70% phosphorous, 14.12% available phosphorous.

^h^Sigma–Aldrich Co. (St. Louis, USA), 50‐micron powder, 98%.

^i^Nutri‐Bind Aqua Dry, Adisseo France SAS (Antony, France). Synthetic pellet binder consisting of modified urea (polymethylolcarbamide).

^j^Rovimix 2050 Px Camarões VitMin (SAM) VM25L3 (BR4418A025). DSM Produtos Nutricionais Brasil Ltda. (São Paulo, Brazil). Guarantee levels per kg of product: vitamin A, 2,996,333 IU; vit. D3, 1,080,066 IU; vit. E, 22,344,50 mg; vit. K3, 7350 mg; vit. B1, 14,560 mg; vit. B2, 13,200 mg; vit. B5, 45,070 mg; vit. B6, 14,560 mg; vit. B12, 7.63 mg; folic acid, 1870 mg; nicotinic acid, 26,350 mg; biotin, 381 mg; inositol, 83,000 mg; Cu, 11,000 mg; I, 500 mg; Mn, 5000 mg; Se, 134 mg; Zn, 31,000 mg; and Co, 1350 mg.

^k^Cholesterol SF (Dishman Netherlands B.V., Veenendaal, The Netherlands). Minimum of 91% active cholesterol.

^l^60% of active choline. Zouping Jujia Choline Industrial Co., Ltd. (Handian Industrial Zone, Zouping County, Shandong, China).

^m^Rovimix Stay *C* 35. Minimum of 35% phosphorylated vitamin C activity. DSM Nutritional Products AG (Schweiz, Switzerland).

^n^MetAMINO, Evonik Operations GmbH (Hanau, Germany). DL‐methionine, feed grade 99%.

^o^Tryptophan not analyzed.

Five experimental diets were formulated to evaluate the effects of graded replacement of SLM and PBM with PV on the growth performance of juvenile *P. vannamei* (Table 3). A CTL diet was formulated without PV and contained SLM (10.00%, as‐is basis) and PBM (8.00%) as the primary animal protein sources. Four additional diets were formulated by progressively replacing the inclusion levels of SLM and PBM with PV at 25%, 50%, 75%, and 100% replacement levels, respectively, referred to as PV25, PV50, PV75, and PV100 (Table 3). Accordingly, PV was included at 5.65%, 11.30%, 16.95%, and 22.60% of the diet in PV25, PV50, PV75, and PV100, respectively, while the inclusion levels of SLM and PBM were proportionally reduced and completely removed in the PV100 diet. Cassava starch levels were gradually reduced to accommodate increasing PV inclusion, while salmon oil and soy lecithin levels were incrementally adjusted to maintain an adequate lipid supply. Squid meal and krill meal were included at constant levels across all diets to support feed palatability and attractability. To maintain an adequate AA balance among diets, crystalline L‐Lys, DL‐Met, and L‐threonine were supplemented at increasing levels with higher PV inclusion. The experimental diets were formulated to be isonitrogenous and isolipidic, with mean CP levels of 35.75 ± 1.01% (± standard deviation [SD]), 8.45 ± 0.13% EE, and 16.47 ± 0.22 MJ/kg GE (Table 3).

**Table 3:** Ingredient and amino acid composition (% of diet, as‐is basis) of the control diet (CTL, without *Paecilomyces variotii*) and experimental diets formulated with graded replacement of salmon by‐product meal (SLM) and poultry by‐product meal (PBM) by *P. variotii* (PV).

Ingredient	Diets/ingredient composition (%, as‐is)				
	CTL	PV25	PV50	PV75	PV100
Soybean meal^a^	36.79	36.79	36.79	36.79	36.79
Wheat flour^a^	24.40	24.40	24.40	24.40	24.40
Salmon by‐product meal (SLM)^b^	10.00	7.50	5.00	2.50	—
Poultry by‐product meal (PBM)^c^	8.00	6.00	4.00	2.00	—
*P. variotii^b^*	—	5.65	11.30	16.95	22.60
Cassava starch	6.00	5.00	3.89	1.98	0.06
Salmon oil^a^	3.79	3.99	4.19	4.38	4.58
Krill meal^a^	1.50	1.50	1.50	1.50	1.50
MSP^a^	1.75	0.82	—	—	—
Potassium chloride	1.39	1.26	1.12	0.98	0.85
Magnesium sulfate	1.26	1.28	1.30	1.32	1.34
Squid meal^d^	1.00	1.00	1.00	1.00	1.00
Soy lecithin oil	0.96	1.14	1.32	1.50	1.68
Calcium carbonate	0.96	1.35	1.73	2.09	2.46
Salt	0.67	0.70	0.74	0.77	0.81
Synthetic binder^a^	0.50	0.50	0.50	0.50	0.50
Vitamin‐mineral premix^a^	0.30	0.30	0.30	0.30	0.30
L‐lysine, 54.6%^e^	0.42	0.49	0.55	0.62	0.69
DL‐methionine, 99%^a^	0.10	0.13	0.16	0.20	0.23
Choline chloride, 60%^a^	0.10	0.10	0.10	0.10	0.10
Stay C, 35%^a^	0.10	0.10	0.10	0.10	0.10
L‐threonine, 98.5%^f^	0.003	0.005	0.008	0.011	0.013
Proximate composition (%, as‐is)					
Dry matter	88.66	87.61	88.57	89.90	89.16
Crude protein	35.62	37.19	36.27	34.99	34.70
Ether extract	8.47	8.24	8.44	8.53	8.58
Crude fiber	1.85	2.11	2.71	1.89	3.25
Ash	9.32	8.63	8.25	8.27	8.13
Gross energy (MJ/kg)	16.46	16.22	16.69	16.28	16.69
Calcium	1.04	1.04	1.03	1.14	1.26
Phosphorous	1.06	0.88	0.96	0.77	0.77
Essential amino acids (EAAs)					
Arginine	2.00	2.02	2.04	2.06	2.02
Histidine	0.83	0.80	0.80	0.79	0.71
Isoleucine	1.36	1.35	1.36	1.36	1.34
Leucine	2.25	2.25	2.26	2.23	2.18
Lysine	2.05	2.06	2.11	2.13	2.11
Methionine (Met)	0.57	0.61	0.60	0.60	0.59
Met + cysteine^a^	0.99	0.97	0.95	0.95	0.89
Phenylalanine	1.44	1.43	1.42	1.43	1.39
Threonine	1.24	1.24	1.25	1.23	1.23
Tryptophan	0.24	0.28	0.30	0.25	0.31
Valine	1.47	1.47	1.47	1.47	1.44
Essential amino acids (EAAs)					
Alanine	1.58	1.57	1.55	1.53	1.49
Aspartic acid	2.97	2.95	2.92	2.85	2.53
Cysteine	5.33	5.25	5.21	5.12	4.87
Glycine	0.42	0.36	0.35	0.35	0.30
Glutamic acid	1.90	1.79	1.66	1.54	1.38
Hydroxyproline	0.31	0.24	0.17	0.01	0.01
Proline	1.82	1.75	1.68	1.64	1.52
Serine	1.45	1.43	1.44	1.43	1.36
Taurine	0.15	0.11	0.08	0.06	0.01
Tyrosine	0.98	1.02	0.98	1.01	0.97
∑EAA	13.45	13.51	13.61	13.55	13.32
∑NEAA	16.91	16.47	16.04	15.54	14.44
∑EAA + NEAA	30.36	29.98	29.65	29.09	27.76

*Note:* The PV25, PV50, PV75, and PV100 diets correspond to 25%, 50%, 75%, and 100% replacement levels, respectively, according to the experimental formulation strategy. Replacement levels refer to the proportional reduction of SLM and PBM inclusion in the formulation and their replacement with graded inclusions of *P. variotii*. Diets were used to evaluate the growth performance of juvenile *P. vannamei*.

^a^Composition shown in Table 2.

^b^Composition shown in Table 1.

^c^BRF S.A. (Capinzal, Brazil). 4.08% moisture, 69.79% CP, 13.66% EE, 1.40% CF, 8.48% ash, 1.49% Met, 4.36% Lys, and 2.35% M + C.

^d^Peruvian Sea Food S.A. (Paita, Peru). 6.81% moisture, 80.18% CP, 4.28% EE, 2.37% CF, 7.44% ash, 2.25% Met, 5.03% Lys, and 3.08% M + C.

^e^Biolys, Evonik Operations GmbH (Hanau, Germany). L‐lysine 54.6%.

^f^ThreAMINO, Evonik Operations GmbH (Hanau, Germany). L‐threonine 98.5%.

Experimental diets were manufactured using a laboratory‐scale pelleting system (model EX‐Micro, Exteec Máquinas, Ribeirão Preto, São Paulo, Brazil). All dry ingredients, except wheat flour, were ground in a hammer mill (model 280, Máquinas Vieira Indústria e Comércio Ltda., Tatuí, Brazil) to a particle size <300 µm, with 68%, 28%, and 4% of particles retained at 300, 250, and <250 µm, respectively. Dry and liquid ingredients were weighed using a two‐decimal electronic balance (model PR4202BR/E, Ohaus Corporation, Tamboré, Brazil). Feed additives and chromium (Cr) (III) oxide (for digestibility diets) were initially premixed with a 1‐kg subsample of the dry macroingredients in a Y‐mixer (model MA201/5MO, Marconi Equipamentos para Laboratórios Ltda., Piracicaba, Brazil) to ensure homogeneous dispersion and subsequently incorporated into a planetary mixer (model AR‐25, G. Paniz, Caixas do Sul, Brazil) containing the remaining dry and liquid ingredients. During mixing, tap water was gradually added at 5 L per 15 kg of feed mash, increasing mash moisture to ~34%. The resulting dough was forced through a rigid plastic mesh to form small fragments, which were then fed into the pelleting machine operated at 95°C. Pellets were cut to ~1.8 mm in diameter and 4.4 mm in length, steam‐conditioned for 3 min, and dried in a forced‐air convection oven (model MA‐035/3, Marconi Equipamentos para Laboratório Ltda., Piracicaba, Brazil) until moisture content reached 10.0%–12.0%. Dried pellets were stored at 16°C until use.

### 2.4. Rearing Systems

To determine in vivo digestibility, 528 shrimp of 8.74 ± 0.75 g body weight (BW) were stocked in 44 rectangular polypropylene tanks (61 L; 31.0 × 35.5 × 55.5 cm; 0.19 m^2^ bottom area). The system operated under a partial water recirculation regime at a rate of 15.65–21.81 L/h (26.1%–36.4% of tank volume per h). Water quality parameters (pH, temperature, and salinity) were measured once daily at 09:00 h in all tanks. Mean (± SD) salinity, pH, and temperature were 14.0 ± <0.01 g/L (*n* = 531), 7.4 ± 0.3 (*n* = 531), and 28.4 ± 0.8°C (*n* = 531), respectively.

Shrimp growth performance was carried out in indoor tanks under clear water conditions. The rearing system consisted of independent circular tanks (0.5 m^3^; 0.46 × 0.85 m; height × diameter; 0.57 m^2^ bottom area) housed under a roofed facility and exposed to a 12‐h artificial photoperiod starting at 05:30 h. Tanks operated under a continuous water recirculation regime at 1.9 ± 0.6 L/min (23% of tank volume/h/). Mean (± SD) salinity, pH, and temperature reached 15.7 ± 1.4 g/L (*n* = 3000 readings), 8.0 ± 0.2 (*n* = 3000), and 28.5 ± 0.6°C (*n* = 3000), respectively.

All tanks were equipped with a perforated lid attached to the top to avoid shrimp escape. Continuous aeration was provided through 0.5‐m air diffusers positioned near the tank bottom and opposite to the feed delivery point to maintain dissolved oxygen near saturation. Tanks were filled with sand‐filtered and chemically disinfected (30 ppm of active calcium hypochlorite) seawater. Shrimp were reared for 75 days in the digestibility system and 78 days in the growth performance system, respectively.

### 2.5. Shrimp Feeding and Feces Collection

For the growth performance, shrimp were fed exclusively using feeding trays (14.3 × 3.5 cm, diameter × height). At each feeding time, trays were inspected for uneaten feed, which was collected, weighed, and discarded. Feed delivery and collection of uneaten feed, when present, were performed at the following times: 1st meal, 07:00–10:00; 2nd meal, 10:00–13:00; 3rd meal, 13:00–16:00; and 4th meal, 16:00–07:00 h.

The daily feed allowance was calculated according to the equation MM = 0.0931× BW^0.6200^, where MM represents the maximum feed intake per day for an individual shrimp with a given BW [26]. A previous study indicated that MM could be restricted by up to 28.8% without negatively affecting shrimp growth performance [27]. Accordingly, daily feed rations were reduced by 30% across all treatments to improve FCR CTL. During the first 2 weeks after stocking, shrimp were fed assuming a fixed daily weight gain (DWG) of 0.05 g/shrimp. Starting on day 14 of rearing and subsequently at biweekly intervals, five shrimp per tank were sampled to estimate mean BW gain. Between sampling days, feed rations were adjusted assuming the average DWG achieved during the previous rearing interval for each individual tank while maintaining a fixed daily reduction in shrimp survival across all dietary treatments. Dead shrimp were not replaced throughout the experimental period.

In the digestibility assay, shrimp feeding and feces collection followed the methods described by Vieira et al. [28]. Shrimp were fed to apparent satiation at 07:00, 10:00, 13:00, and 16:00 h using feeding trays (9.75 cm diameter). Feed residues were removed before feces collection to minimize contamination. Daily feed amounts were adjusted based on the quantity of uneaten feed collected from the trays. When present, uneaten feed was collected, oven‐dried in a convection dryer, weighed, and discarded. Feces were collected by siphoning four times daily, gently rinsed with distilled water to remove salt residues, and stored at −23°C until analysis. All fecal samples were freeze‐dried prior to chemical analysis.

### 2.6. Growth Performance and ADCs

At harvest, all live shrimp were sacrificed by thermal shock through immersion in a mixture of crushed ice and seawater (2:1 ice‐to‐seawater ratio) until no movement was observed. Their growth performance was determined by individually weighing all shrimp. Final shrimp survival (%) was calculated as (POPf ÷ POPi) × 100, where POPi is the number of shrimp stocked and POPf is the number of shrimp harvested. The weekly weight gain (g/week) was determined as [(BWf – BWi) ÷ *t*] × 7, where BWi and BWf represent the initial and final mean shrimp BWs (g), respectively, and *t* is the number of rearing days. The gained shrimp yield (g/m^2^) was calculated as (BIOf – BIOi) ÷ tank bottom area (m^2^), where BIOi and BIOf are the initial and final shrimp biomasses per tank (g). The FCR was obtained by dividing the total feed input (g, as‐is basis) per tank by the total shrimp biomass gained (g, as‐is). The apparent feed intake (AFI, g/shrimp) was determined as the total amount of feed delivered (g, dry matter [DM] basis) divided by the number of stocked shrimp per tank.

ADCs for CP and AAs of PV and SLM were determined from the concentrations of Cr and each respective nutrient/energy in the experimental diets and shrimp feces according to the equation described by Cho et al. [29]:

(1) ADC=100−100%Crd %Crf×%Nf %Nd,

where ADC = ADC of CP and AAs; Cr*d* = concentration (%) of Cr in the diet; Cr*f* = concentration (%) of Cr in shrimp feces; N*d* = concentration (%) of CP and AAs in the test diets; and N*f* = concentration (%) of CP and AA in shrimp feces.

Subsequently, the digestibility of CP and AAs of each individual test ingredient was calculated [30]:

(2) ADCtest ing.=ADCtest diet+ADCtest diet− ADCref. diet×0.7×Dref0.3×Ding.,

where ADC_test ing._ = ADC of CP and AAs in the test ingredients (in %); DC_test diet_ = ADC of CP and AAs in the test diets (in %); ADC_ref. diet_ = ADC of CP and AAs in the REF diet (in %); *D*_ref_ = concentration (%) of CP and AAs in the REF diet; and *D*_ing._ = concentration (%) of CP and AAs in the test ingredients.

### 2.7. Nutrient and Energy Deposition in Shrimp Whole Body

At stocking and harvest of the growth‐performance system, live shrimp were sampled for proximate composition analyses, including DM, CP, EE, CF, GE, ash, calcium, and phosphorus. To calculate nutrient and energy retention efficiency, an initial subsample of 40 live shrimp was collected to determine the DM content. After excess surface water was removed, shrimp were individually weighed and dried in a forced‐air oven at 105°C for 24 h. At stocking, a total of 1 kg of shrimp were collected and freeze‐dried. At harvest, three composite samples per dietary treatment were prepared, each consisting of shrimp collected from three to four culture tanks, with 15 shrimp sampled per tank. Whole shrimp (head‐on and shell‐on) were freeze‐dried, ground, and homogenized. Nutrient and energy deposition efficiency (%) was calculated as: {[(final BW × nutrient/energy content at harvest) – (initial BW × nutrient/energy content at stocking) ÷ (dietary nutrient/energy content x AFI)]} × 100.

### 2.8. Chemical Analyses

Chemical analyses followed standard methods [31]. DM was determined in a convection oven at 105°C for 24 h (AOAC 934.01). CP was analyzed by the Kjeldahl method for nitrogen determination (AOAC 984.13). EE was determined by rapid high‐temperature solvent extraction using petroleum ether, following the AOCS Official Procedure Am 5‐04 [32]. Ash content was determined by incineration in a muffle furnace at 600°C for 2 h (AOAC 942.05). CF was determined by the enzymatic–gravimetric method (AOAC 992.16). GE was measured by bomb calorimetry (AOAC 983.23) using an IKA C5000 calorimeter (IKA‐Werke GmbH & Co. KG, Staufen, Germany). AA content in diets and feces samples was analyzed by wet chemistry using ion‐exchange chromatography [33, 34]. To determine total Cr concentrations in shrimp feces and experimental diets used to assess digestibility, samples (0.200 g) were digested with 2 mL of HNO_3_ (65%), 2 mL of H_2_O_2_ (30%), and 4 mL of Milli‐Q water using an Ethos UP microwave digestion system (Milestone Srl, Sorisole, Italy). Cr concentrations in the digested samples were measured in triplicate by inductively coupled plasma triple quadrupole mass spectrometry (ICP‐QQQ; Agilent 8800 ICP‐QQQ, Agilent Technologies, Tokyo, Japan). Method accuracy and precision of the digestion and ICP‐QQQ analytical procedures were verified using Certified REF Materials V‐10 (hay powder), NRC DOLT‐4 (fish liver), and C4001a (internal CRM).

### 2.9. Statistical Analysis

Statistical analyses were performed using IBM SPSS Statistics version 23.0 (SPSS Inc., Chicago, IL, USA). Homogeneity of variance was examined for all data by using the Bartlett‐Box F and Cochran’s C tests. Kurtosis and skewness and their standard error (i.e., s.e. kurtosis and s.e. skewness) were applied to the data as measures of asymmetry and tests of normality. One‐way analysis of variance (ANOVA) was used to evaluate differences among dietary treatments for shrimp growth performance parameters (final survival, growth, yield, final BW, AFI, and FCR), ADCs (CP and AA), shrimp whole‐body proximate composition at harvest, and nutrient and energy retention efficiency. When significant effects were detected, Tukey’s HSD tests were applied for pairwise comparisons of treatment means. Student’s *t*‐test was used to compare ADCs between the test ingredients (PV and SLM). Statistical significance was set at *p* < 0.05.

## 3. Results

### 3.1. Diet Chemical Composition

In the digestibility diets, ΣEAAs reached 19.03% (%, dry matter [DM] basis) in SLM, 17.31% in PV, and 16.29% in REF (Table 2). Lys levels were 2.78% (SLM), 2.35% (PV), and 2.05% (REF), while Met levels were 1.14%, 0.84%, and 0.92%, respectively. Met plus cysteine reached 1.60% in SLM, 1.37% in PV, and 1.36% in REF. Taurine levels decreased from 0.41% (SLM) to 0.20% (PV) and hydroxyproline from 0.78% to 0.01%. Cr concentrations were similar among diets, with values of 3.44 ± 2.35, 3.40 ± 2.37, and 3.45 ± 2.03 mg/kg (DM basis) for REF, SLM, and PV, respectively.

In the growth‐performance diets, ΣEAA levels remained stable between 13.32% and 13.61% across treatments (Table 3). Lys ranged from 2.05% (CTL) to 2.13% (PV75), Met from 0.57% to 0.61%, and Met plus cysteine from 0.99% to 0.89% as PV inclusion increased from 0% to 100%. Tryptophan varied between 0.24% and 0.31% (coefficient of variation [CV] = 11.05%). Several nonessential AAs (NEAAs) declined with increasing PV inclusion, including glycine (0.42% to 0.30%), glutamic acid (1.90% to 1.38%), proline (1.82% to 1.52%), hydroxyproline (0.31% to 0.01%), and taurine (0.15% to 0.01%). Total NEAAs (ΣNEAAs) decreased from 16.91% (CTL) to 14.44% (PV100) and total AAs (ΣEAA + ΣNEAA) from 30.36% to 27.76%.

### 3.2. ADCs and Growth Performance

During the digestibility trial, shrimp were fed in excess to maximize fecal production. Survival (93.8 ± 9.0%), final BW (30.84 ± 4.84 g), weekly growth (2.06 ± 0.16 g), gained yield (1210 ± 205 g/m^2^), AFI (31.8 ± 0.8 g/shrimp), and FCR (1.64 ± 0.38) did not differ significantly among the REF, PV, and SLM diets (*p* > 0.05). In contrast, all ADCs differed significantly among diets and ingredients (Table 4).

**Table 4:** Apparent digestibility coefficients (ADCs) of the diets and ingredients evaluated in the study.

Nutrients	Apparent digestibility coefficients (ADCs, %)						
	Diet			*p* Sig.	Ingredient		*p* Sig.
	REF	SLM	PV		SLM	*P. variotii*	
Crude protein	88.34 ± 0.25^a^	81.44 ± 0.95^b^	78.94 ± 0.12^c^	<0.0001	72.02 ± 1.96	62.28 ± 0.48	<0.0001
Essential amino acids (EAAs)							
Arginine	94.02 ± 0.23^a^	90.28 ± 0.41^b^	89.99 ± 0.25^b^	<0.0001	84.97 ± 0.91	83.54 ± 0.64	0.001
Histidine	90.48 ± 0.14^a^	84.90 ± 0.59^b^	80.55 ± 1.50^c^	<0.0001	77.05 ± 1.24	55.59 ± 4.59	<0.0001
Isoleucine	91.33 ± 0.35^a^	83.94 ± 0.90^c^	85.68 ± 0.45^b^	<0.0001	71.42 ± 2.17	74.31 ± 1.32	<0.0001
Leucine	92.04 ± 0.30^a^	85.54 ± 0.85^b^	85.64 ± 0.29^b^	<0.0001	74.79 ± 1.99	72.92 ± 0.92	0.004
Lysine	94.30 ± 0.38^a^	91.81 ± 0.61^b^	91.19 ± 0.18^b^	<0.0001	89.35 ± 1.10	86.23 ± 0.66	0.002
Methionine (Met)	94.99 ± 0.32^a^	90.71 ± 0,53^b^	89.42 ± 0.45^c^	<0.0001	85.71 ± 1.05	73.21 ± 1.72	<0.0001
Met + cysteine	93.74 ± 0.33^a^	88.34 ± 0.61^b^	88.10 ± 0.49^b^	<0.0001	81.62 ± 1.38	73.90 ± 1.65	< 0.0001
Phenylalanine	90.08 ± 0.34^a^	82.06 ± 0.53^b^	81.25 ± 0.41^b^	<0.0001	68.68 ± 1.78	61.16 ± 1.34	<0.0001
Threonine	89.29 ± 0.17^a^	81.70 ± 0.69^b^	81.63 ± 0.42^b^	<0.0001	71.97 ± 1.03	69.59 ± 0.96	<0.0001
Valine	89.68 ± 0.40^a^	82.14 ± 0.74^b^	82.16 ± 0.25^b^	<0.0001	71.36 ± 1.51	68.27 ± 0.89	<0.0001
Nonessential amino acids (NEAAs)							
Alanine	90.62 ± 0.22^a^	86.00 ± 0.51^b^	84.04 ± 0.06^c^	<0.0001	81.14 ± 0.78	73.43 ± 0.34	<0.0001
Aspartic acid	96.75 ± 0.32^a^	94.55 ± 0.68^b^	93.59 ± 0.16^c^	<0.0001	91.62 ± 1.14	86.91 ± 0.72	<0.0001
Glutamic acid	96.45 ± 0.26^a^	93.38 ± 0.43^b^	92.16 ± 0.12^c^	<0.0001	87.89 ± 1.56	79.43 ± 0.78	<0.0001
Cysteine	91.13 ± 0.38^a^	82.45 ± 0.54^c^	86.04 ± 0.70^b^	<0.0001	70.04 ± 2.50	73.39 ± 2.28	0.009
Glycine	91.34 ± 0.05^a^	88.50 ± 0.62^b^	81.94 ± 0.21^c^	<0.0001	86.10 ± 0.79	57.60 ± 0.67	<0.0001
Hydroxyproline	95.46 ± 6.55^a^	99.31 ± 1.17^a^	42.28 ± 0.23^b^	<0.0001	100 ± 3.21	—	—
Proline	92.21 ± 0.16^a^	86.97 ± 0.49^b^	84.27 ± 0.16^c^	<0.0001	79.25 ± 0.64	59.47 ± 0.73	<0.0001
Serine	88.56 ± 0.12^a^	80.90 ± <0.001^b^	78.89 ± 0.08^c^	<0.0001	70.33 ± 1.00	60.26 ± 0.29	<0.0001
Taurine	98.82 ± <0.001^a^	94.72 ± 0.28^c^	96.96 ± 0.01^b^	<0.0001	92.04 ± 0.02	—	—
Tyrosine	89.70 ± 0.59^a^	81.93 ± 0.48^b^	91.37 ± 0.49^b^	<0.0001	68.98 ± 2.19	65.11 ± 1.60	0.001
∑EAA	91.79 ± 0.29^a^	85.98 ± 0.02^b^	85.73 ± 0.31^b^	<0.0001	77.69 ± 1.36	73.47 ± 0.95	<0.0001
∑NEAA	93.30 ± 0.09^a^	89.10 ± 0.87^c^	86.55 ± 0.09^b^	<0.0001	83.97 ± 1.01	71.37 ± 0.30	<0.0001
∑EAA + NEAA	92.63 ± 0.15^a^	87.72 ± 0.62^b^	86.17 ± 0.14^c^	<0.0001	81.33 ± 1.14	72.31 ± 0.46	<0.0001

*Note:* Values are presented as mean (± standard deviation) of three fecal samples from each diet and/or ingredient, obtained from four to five tanks stocked with 12 shrimp each. Identical letters indicate no significant statistical difference among diets at *α* = 0.05 according to Tukey’s HSD test (diets) or Student’s *t*‐test (ingredients).

For CP, ADCs were 88.34 ± 0.25% (REF), 81.44 ± 0.95% (SLM), and 78.94 ± 0.12% (PV) at the diet level (*p* < 0.0001) and 72.02 ± 1.96% (SLM) and 62.28 ± 0.48% (PV) at the ingredient level (*p* < 0.0001). For essential AAs (EAAs), diet ADCs were consistently high in the REF diet, intermediate in SLM, and low in PV. Lys ADCs were 94.30 ± 0.38% (REF), 91.81 ± 0.61% (SLM), and 91.19 ± 0.18% (PV), while Met ADCs reached 94.99 ± 0.32%, 90.71 ± 0.53%, and 89.42 ± 0.45%, respectively (all *p* < 0.0001). Histidine showed the lowest ADC among EAAs, with 90.48 ± 0.14% (REF), 84.90 ± 0.59% (SLM), and 80.55 ± 1.50% (PV). At the ingredient level, ADCs for EAAs were generally higher for SLM than PV, including Lys (89.35 ± 1.10% vs. 86.23 ± 0.66%), Met (85.71 ± 1.05% vs. 73.21 ± 1.72%), and histidine (77.05 ± 1.24% vs. 55.59 ± 4.59%).

For NEAAs, REF diet ADCs ranged from 88.56 ± 0.12% (serine) to 98.82 ± <0.001% (taurine), whereas PV diet ADCs ranged from 42.28 ± 0.23% (hydroxyproline) to 96.96 ± 0.01% (taurine) (*p* < 0.0001). Glycine ADCs decreased from 91.34 ± 0.05% (REF) to 81.94 ± 0.21% (PV). At the ingredient level, glycine ADCs were 86.10 ± 0.79% (SLM) and 57.60 ± 0.67% (PV), while proline ADCs were 79.25 ± 0.64% and 59.47 ± 0.73%, respectively.

Summed ADCs for ΣEAA were 91.79 ± 0.29% (REF), 85.98 ± 0.02% (SLM), and 85.73 ± 0.31% (PV) at the diet level and 77.69 ± 1.36% (SLM) and 73.47 ± 0.95% (PV) at the ingredient level (*p* < 0.0001). For ΣNEAA, diet ADCs were 93.30 ± 0.09% (REF), 89.10 ± 0.87% (SLM), and 86.55 ± 0.09% (PV), while ingredient ADCs were 83.97 ± 1.01% (SLM) and 71.37 ± 0.30% (PV). Total AA ADCs (ΣEAA + ΣNEAA) reached 92.63 ± 0.15% (REF), 87.72 ± 0.62% (SLM), and 86.17 ± 0.14% (PV) at the diet level and 81.33 ± 1.14% (SLM) and 72.31 ± 0.46% (PV) at the ingredient level (*p* < 0.0001).

In the growth performance trial, initial BW was similar among treatments (1.00 ± 0.17–1.02 ± 0.17 g; *p* = 0.389; Table 5). Final survival remained high across treatments (93.17 ± 5.85–97.67 ± 2.63%) and did not differ significantly (*p* = 0.124). Weekly growth averaged between 1.02 ± 0.10 and 1.06 ± 0.07 g/week (*p* = 0.986), while gained yield reached 1127 ± 90–1209 ± 78 g/m^2^ (*p* = 0.262). AFI varied from 14.4 ± 0.5 to 14.8 ± 0.7 g/shrimp (*p* = 0.250), and FCR remained between 1.30 ± 0.05 and 1.37 ± 0.12 (*p* = 0.392).

**Table 5:** Growth performance of juvenile *P. vannamei* reared in 50 indoor tanks (0.5 m^3^) under 105 animals/m^2^ for 78 days.

Growth performance	Diets					*p* ANOVA
	CTL	PV25	PV50	PV75	PV100	
Initial body weight (g)	1.02 ± 0.17	1.02 ± 0.17	1.01 ± 0.17	1.00 ± 0.17	1.02 ± 0.17	0.389
Final body weight (g)	12.80 ± 0.72^a^	12.57 ± 1.09^ab^	12.58 ± 0.40^ab^	12.57 ± 0.81^ab^	12.31 ± 1.10^b^	0.001
Final survival (%)	97.67 ± 2.63	94.82 ± 5.36	93.17 ± 5.85	95.00 ± 3.51	97.41 ± 3.02	0.124
Growth (g/week)	1.06 ± 0.07	1.04 ± 0.10	1.04 ± 0.04	1.04 ± 0.07	1.02 ± 0.10	0.986
Gained yield (g/m^2^)	1.209 ± 78	1.147 ± 78	1.127 ± 90	1.151 ± 79	1.155 ± 87	0.262
AFI^1^ (g/shrimp)	14.8 ± 0.4	14.7 ± 0.5	14.5 ± 0.4	14.4 ± 0.5	14.8 ± 0.7	0.250
FCR^2^	1.30 ± 0.05	1.36 ± 0.07	1.37 ± 0.12	1.32 ± 0.05	1.35 ± 0.07	0.392

*Note:* Values are presented as mean (± standard deviation) of 10 culture tanks. Values presented as means (± standard deviation). Similar letters indicate nonstatistically significant differences according to Tukey’s HSD test (*p* < 0.05).

^1^Apparent feed intake.

^2^Feed conversion ratio.

Final BW differed significantly among dietary treatments (*p* = 0.001; Table 5).

Shrimp fed the CTL diet (12.80 ± 2.02 g) and diets with up to 75% replacement of SLM and PBM with PV (12.57 ± 1.74 g) exhibited similar BW. Shrimp fed the diet with 100% replacement exhibited a lower final BW (12.31 ± 1.89 g) compared with the CTL diet (*p* < 0.0001).

### 3.3. Whole‐Body Composition and Nutrient/Energy Retention

Whole‐body proximate composition of shrimp at harvest was largely unaffected by dietary treatment (*p* > 0.05; Table 6). CP content remained stable across diets, varying narrowly between 78.01 ± 0.63% and 78.48 ± 0.68% DM basis (*p* = 0.927). Likewise, EE (5.06%–6.15%; *p* = 0.098), CF (4.89%–5.92%; *p* = 0.296), GE (18.61–19.97 MJ/kg; *p* = 0.291), calcium (2.59%–2.80%; *p* = 0.259), and phosphorus (1.19%–1.33%; *p* = 0.381) did not differ significantly among treatments. In contrast, ash content differed among diets (*p* = 0.023), with shrimp fed the PV100 diet (12.96 ± 0.32%) showing higher ash levels than those fed PV25 (11.71 ± 0.03%), while the remaining treatments were intermediate.

**Table 6:** Proximate composition of whole shrimp and body retention efficiency of nutrients and energy at the end of the growth performance trial as a function of dietary treatment.

Nutrients/energy	Proximate composition (%, DM basis)					
	CTL	PV25	PV50	PV75	PV100	ANOVA *p*
Crude protein	78.21 ± 0.35	78.42 ± 0.003	78.01 ± 0.63	78.25 ± 1.04	78.48 ± 0.68	0.927
Ether extract	5.90 ± 0.41	5.60 ± 0.30	5.90 ± 0.42	6.15 ± 0.51	5.06 ± 0.47	0.098
Crude fiber	5.24 ± 0.40	5.92 ± 0.34	5.19 ± 0.29	4.89 ± 0.38	4.92 ± 0.87	0.296
Gross energy (MJ/kg)	19.27 ± 0.13	19.24 ± 0.33	19.97 ± 1.47	18.86 ± 0.31	18.61 ± 0.29	0.291
Ash	12.09 ± 0.15^ab^	11.71 ± 0.03^a^	12.21 ± 0.40^ab^	12.33 ± 0.47^ab^	12.96 ± 0.32^b^	0.023
Calcium	2.60 ± 0.19	2.80 ± 0.14	2.59 ± 0.05	2.65 ± 0.07	2.78 ± 0.14	0.259
Phosphorous	1.33 ± 0.18	1.23 ± 0.01	1.19 ± 0.01	1.28 ± 0.04	1.33 ± 0.03	0.381

	**Body retention efficiency (%)**					

Crude protein	34.99 ± 1.55^b^	32.48 ± 2.05^a^	34.30 ± 0.90^b^	37.17 ± 1.99^c^	35.19 ± 2.12^b^	<0.0001
Ether extract	10.96 ± 0.85^bc^	10.28 ± 0.79^b^	11.01 ± 0.76^c^	11.88 ± 1.09^d^	8.90 ± 0.95^a^	<0.0001
Crude fiber	47.00 ± 3.70^c^	45.24 ± 3.33^c^	31.81 ± 1.73^b^	44.68 ± 3.74^c^	24.52 ± 4.02^a^	<0.0001
Gross energy	18.67 ± 083^bc^	18.27 ± 1.18^b^	19.15 ± 1.35^c^	19.24 ± 1.05^c^	17.31 ± 1.07^a^	<0.0001
Ash	20.05 ± 0.95^a^	20.18 ± 1.33^a^	22.91 ± 0.91^b^	24.08 ± 1.54^c^	24.21 ± 1.59^c^	<0.0001
Calcium	38.51 ± 3.16^bc^	40.44 ± 3.12^d^	39.09 ± 1.52^cd^	37.03 ± 2.07^b^	32.60 ± 2.38^a^	<0.0001
Phosphorous	20.23 ± 2.64^a^	21.81 ± 1.36^b^	19.99 ± 0.52^a^	27.97 ± 1.66^c^	27.21 ± 1.69^c^	<0.0001

*Note:* Proximate composition values are presented as the mean (± standard deviation) of three samples composed of 36 to 48 shrimp collected from three to four culture tanks. Similar letters indicate nonstatistically significant differences among dietary treatments at the *α* = 0.05 level according to Tukey’s HSD test.

Marked differences were observed in body retention efficiency of nutrients and energy (Table 6). Protein retention efficiency differed significantly among diets (*p* < 0.0001), with values of 34.99 ± 1.55% (CTL), 32.48 ± 2.05% (PV25), 34.30 ± 0.90% (PV50), 37.17 ± 1.99% (PV75), and 35.19 ± 2.12% (PV100). Shrimp fed PV75 exhibited higher protein retention than those fed PV25, while the remaining treatments were statistically similar.

Lipid retention efficiency ranged from 8.90 ± 0.95% (PV100) to 11.88 ± 1.09% (PV75) (*p*  < 0.0001). Shrimp fed PV100 showed lower lipid retention than all other treatments, whereas PV75 resulted in the highest lipid retention. Energy retention efficiency followed a similar pattern, with values of 17.31 ± 1.07% (PV100), 18.27%–18.67% (CTL and PV25), and 19.15%–19.24% (PV50 and PV75; *p* < 0.0001).

Retention of mineral fractions also differed significantly among diets. Ash retention efficiency increased progressively from 20.05 ± 0.95% (CTL) and 20.18 ± 1.33% (PV25) to 24.08%–24.21% (PV75 and PV100; *p* < 0.0001). Calcium retention efficiency decreased at the highest PV inclusion, with shrimp fed PV100 showing 32.60 ± 2.38%, compared with 38.51%–40.44% in the other treatments (*p* < 0.0001). Phosphorus retention efficiency was lowest in CTL and PV50 (≈20%), intermediate in PV25 (21.81 ± 1.36%), and highest in PV75 and PV100 (27.97 ± 1.66% and 27.21 ± 1.69%, respectively; *p* < 0.0001).

## 4. Discussion

The present study provides a comprehensive evaluation of a fungal biomass (PV), produced from ethanol stillage, as a partial or total replacement for conventional animal protein sources in diets for juvenile *P. vannamei*. By integrating growth performance, in vivo digestibility, and whole‐body nutrient retention data, the results allow a comprehensive assessment of both the nutritional value and the practical inclusion limits of this ingredient in shrimp feeds.

Microbial proteins have been widely evaluated in aquaculture, particularly in fish [5], but several yeast‐, bacterial‐, fungal‐, and microalgal‐based products have also been tested in shrimp diets at various dietary inclusion levels [12, 15, 16, 35], primarily to reduce reliance on fish meal without compromising growth performance. In the present study, overall shrimp performance was largely maintained across diets containing graded levels of PV, as evidenced by similar survival, weekly growth rate, yield, AFI, and FCR. These results indicate that PV can replace a substantial proportion of SLM and PBM without impairing general production metrics under controlled clear water conditions.

The absence of differences in AFI also suggests that feed acceptance was not a major limiting factor in this study. This outcome is likely associated with the constant inclusion of squid meal and krill meal across all diets, ingredients known to enhance feed attractiveness in penaeid shrimp [27, 36]. Therefore, potential palatability limitations associated with PV inclusion were likely mitigated by the dietary formulations employed. Gaudhaman et al. [8] reported that rainbow trout (*O. mykiss*) did not exhibit reduced feed intake when PEKILO, derived from PV, was included in a diet at 30%. However, other microbial sources, such as bacterial proteins derived from *Methylobacterium extorquens*, have been associated with reduced palatability in rainbow trout, even at relatively low inclusion levels (10% of the diet), despite being used in formulations containing up to 30% fish meal [37].

In the present study, most performance indicators were similar across treatments, but final BW declined when shrimp were fed a diet containing 22.60% PV, corresponding to the complete replacement of SLM and PBM. This pattern suggests that high dietary inclusion levels of PV may impose nutritional constraints. In contrast, shrimp growth performance was not affected when PV was included at levels up to 16.95%, equivalent to a 75% replacement of SLM and PBM. These findings are consistent with Hooft et al. [21], who also reported no negative effects on final BW, growth rate, or feed intake in Atlantic salmon, *S. salar*, fed diets containing up to 15.90% (as‐is) PV protein. However, it is important to note that, unlike the present study, Hooft et al. [21] replaced soy protein concentrate and wheat gluten meal with PV, while fish meal inclusion was only slightly reduced, from 34.00% to 31.50%. Hardy et al. [37], who evaluated a bacterial protein derived from *M. extorquens* for rainbow trout, emphasized that methylotrophic bacterium performance is strongly dependent on which ingredients are replaced. In their study, *M. extorquens* partially replaced soybean meal, while fish meal inclusion was maintained at 30%, allowing greater tolerance to the microbial ingredient. Therefore, complete replacement of high‐quality animal proteins (SLM and PBM) by PV appears to be more restrictive than partial replacement of plant proteins, such as soybean meal.

The digestibility results provide a mechanistic explanation for the findings on shrimp growth performance. For SLM, protein ADC values (72.02 ± 1.96%) were lower than those reported by Vieira et al. [28] and Guo et al. [38], who reported protein ADCs of 78.9% and 90.9%, respectively. Lower protein ADC values for SLM may be attributed to differences in ingredient processing and quality, composition of the test diet, analytical methodology, and shrimp physiological and rearing conditions relative to those used in previous studies. For PV, both protein and AA digestibility were markedly lower than that of SLM. This response is likely consistent with the compositional characteristics of fungal biomass, which contains indigestible cell‐wall polysaccharides (chitin, β‐glucans, and α‐glucans), which dilute digestible energy and limit protein and AA availability [5, 8]. These fractions can reduce nutrient utilization efficiency when PV is included at high levels. Similar to our findings, Gaudhaman et al. [8] reported that in rainbow trout (*O. mykiss*), fungal biomass exhibited the highest protein ADC (86.5%) among other filamentous fungi (*Aspergillus oryzae*, *Rhizopus oligosporus*, and *Rhizopus delemar*) but still lower than typical fish meal values (91.2%).

Comparable patterns have been reported in Atlantic salmon, *S. salar*. Hooft et al. [21] evaluated diets containing progressive inclusion levels of PV (0, 4.04%, 7.95%, and 15.90% of the diet, as‐is) and observed linear reductions in the ADCs of CP, GE, and most EAAs and NEAAs with increasing inclusion. However, no significant reductions in digestibility were detected when PV replaced up to ~10% of dietary CP (corresponding to 4.04% dietary inclusion), indicating that only low inclusion levels maintained nutrient digestibility for Atlantic salmon.

While most studies with fish have used PV produced from sugar beet vinasse [6, 8, 39], the present study employed corn ethanol thin stillage instead. Vielma et al. [39] reported that the production substrate markedly influences the digestibility of PV. In rainbow trout, authors reported protein ADC values of 88.6% and 74.5% for PV produced using by‐products from spent sulfite liquor and sugar beet vinasse, respectively, whereas a substantially lower protein digestibility (60.5%) was observed for the yeast *Rhodotorula babjevae*. More recently, Abimorad et al. [40] showed that PV biomass produced from corn ethanol thin stillage exhibited high protein (85.47 ± 1.68%) and AA (>88% for all AAs) digestibility in Nile tilapia (*Oreochromis niloticus*) and supported growth performance at dietary inclusion levels of up to 20%, further confirming the feasibility of alternative ethanol‐derived substrates for fungal biomass production.

In our work, the reduced digestibility for PV of specific AAs (Met, histidine, glycine, and proline) is of particular relevance given their roles in shrimp growth and metabolism [41]. Crystalline AAs (L‐Lys, DL‐Met, and L‐threonine) were progressively supplemented in experimental diets as PV inclusion increased. However, the lower ADCs observed for several AAs in PV‐rich diets suggest that their effective bioavailability may have become limiting only at the highest PV level (22.60%). Comparable reductions in digestibility of DM, energy, and protein have been reported in rainbow trout diets containing 20%–30% PV, particularly when additional processing steps such as double extrusion were not applied [22].

Whole‐body proximate composition at harvest was largely unaffected by dietary treatment, indicating that shrimp carcass composition is relatively resilient to moderate changes in dietary protein sources. Despite the lower digestibility coefficients observed for PV relative to SLM, shrimp whole‐body composition remained stable across treatments, likely reflecting the strong physiological regulation of body nutrient deposition when diets remain nutritionally balanced and feed intake is maintained. In the present study, diets were formulated to be isonitrogenous and isolipidic and supplemented with crystalline AAs, which likely contributed to maintaining carcass composition despite the lower ADCs observed for the fungal ingredient. In contrast, nutrient and energy retention efficiencies differed among treatments. Protein retention was highest in shrimp fed the PV75 diet, suggesting that moderate inclusion of PV may have improved the utilization efficiency of absorbed nutrients, whereas very low or very high inclusion levels appear less favorable. Lipid and energy retention efficiencies were reduced in shrimp fed the PV100 diet, consistent with the lower digestibility and energy density of PV, and are consistent with the reduced final BW observed under complete replacement. Mineral retention patterns further highlight the metabolic implications of high PV inclusion. Increased ash and phosphorus retention at higher PV levels may reflect differences in mineral bioavailability or endogenous regulation rather than improved mineral nutrition. Conversely, reduced calcium retention at complete replacement is consistent with the negligible calcium content of PV and reinforces the need for careful mineral balancing when fungal proteins are used extensively in shrimp diets.

## 5. Conclusion

The present study demonstrates that fungal biomass (PV) produced from corn ethanol thin stillage is a nutritionally viable and sustainable alternative protein source for juvenile *P. vannamei* when used as a partial replacement for conventional animal proteins. Inclusion levels of up to 16.95% PV, corresponding to 75% replacement of SLM and PBM, supported satisfactory growth performance, feed utilization, nutrient digestibility, and whole‐body nutrient retention under clear water culture conditions.

In contrast, complete replacement of animal protein sources (22.60% PV) resulted in reduced protein and energy digestibility, lower nutrient retention efficiencies, and a modest but significant reduction in final BW, indicating that high inclusion levels impose nutritional constraints. These limitations appear to be primarily associated with reduced digestibility of protein and specific AAs, particularly Met, as well as the lower energy density and mineral imbalance characteristic of fungal biomass.

Overall, the findings confirm that PV biomass produced from corn ethanol thin stillage can be effectively incorporated into shrimp feeds when inclusion levels are carefully balanced. Further improvements in formulation strategies, AA supplementation, and processing technologies may help overcome digestibility constraints at higher inclusion levels, thereby expanding the practical use of fungal biomass as sustainable protein ingredients in shrimp feeds.

## Funding

This work was fully funded by the FS Indústria de Biocombustíveis Ltda., Brazil.

## Disclosure

The funder had no role in the study design, data collection, data analysis, interpretation of results, manuscript preparation, or the decision to publish.

## Ethics Statement

All experimental procedures involving shrimp were conducted in accordance with applicable national legislation and institutional guidelines for the use of animals in research. Shrimp are aquatic invertebrates and are not covered by Brazilian Federal regulations requiring ethical committee approval for animals of the phylum Chordata, subphylum Vertebrata; therefore, formal approval from an Institutional Animal Care and Use Committee was not required for this study. No anesthetic or surgical procedures were performed. All reasonable measures were taken throughout the experimental period to minimize stress and avoid unnecessary suffering, including appropriate stocking densities, continuous aeration, maintenance of optimal water quality, and regular monitoring of animal health.

## Conflicts of Interest

Luana Quisini Vivan is employed by FS Indústria de Biocombustíveis Ltda., Brazil, which produces fungal single‐cell protein used in this study. The other authors declare no conflicts of interest.

## Data Availability

The data that support the findings of this study are available from the corresponding author upon reasonable request.
